# The Efficacy and Safety of Colpocleisis for Urinary Retention in Elderly Women With Pelvic Organ Prolapse

**DOI:** 10.7759/cureus.98680

**Published:** 2025-12-08

**Authors:** Akihiro Maeda, Shohei Tobu, Maki Kawasaki, Hiroaki Kakinoki, Mitsuru Noguchi

**Affiliations:** 1 Department of Urology, Faculty of Medicine, Saga University, Saga, JPN

**Keywords:** colpocleisis, elderly women, pelvic organ prolapse, urinary retention (ur), voiding difficulty

## Abstract

Objective

This study aimed to evaluate the clinical efficacy and safety of colpocleisis for urinary retention in elderly women with advanced pelvic organ prolapse (POP), with a particular focus on symptom resolution, postoperative voiding function, and perioperative safety.

Methods

This retrospective study included nine elderly women (median age: 79 years) with POP-associated urinary retention who underwent colpocleisis at our institution between January 2019 and December 2024. Colpocleisis was performed only in patients who were not sexually active and did not desire future sexual activity. Urinary retention was defined as the inability to void spontaneously, requiring either intermittent catheterization or placement of an indwelling urethral catheter. All patients underwent a preoperative urodynamic evaluation. Surgical outcomes and the postoperative voiding function were assessed, including the voided volume, maximum urinary flow rate (Qmax), and post-void residual volume (PVR).

Results

Eight of the nine patients (89%) achieved spontaneous voiding postoperatively and were successfully weaned from catheterization. One patient with spinal cord injury continued to require catheterization but experienced easier management and an overall improvement in quality of life. No perioperative complications were observed, and no POP recurrence was noted during a median follow-up of three (range: 1-18) months. The median postoperative voided volume, Qmax, and PVR were 175 mL, 15.9 mL/s, and 20 mL, respectively.

Conclusions

Colpocleisis may represent a safe and effective surgical option in elderly women with POP-related urinary retention. It facilitates spontaneous voiding or simplifies catheter management, contributing to an improved quality of life. Given its minimally invasive, mesh-free nature and short operative time, colpocleisis could be a valuable treatment option for elderly patients with voiding dysfunction who do not wish to preserve sexual function.

## Introduction

Pelvic organ prolapse (POP) is a condition in which the pelvic organs, including the bladder, uterus, and rectum, descend beyond the vaginal opening due to weakening of the pelvic floor support structures. It predominantly affects elderly women [1,2]. POP can cause bladder outlet obstruction (BOO) by compressing the urethra or bending the bladder neck, leading to voiding dysfunction [3,4]. In older patients, additional factors such as age-related decline in bladder contractility, history of pelvic surgery, diabetes mellitus, and spinal disorders may contribute to detrusor underactivity (DU), which can ultimately result in urinary retention [5].

Urinary retention in women is relatively uncommon and lacks well-established diagnostic criteria and standardized treatment algorithms, making clinical management challenging [6]. In patients with POP, clean intermittent self-catheterization is often difficult because of prolapsed organs, and pessary use may be hindered by frequent dislodgement. Therefore, long-term urethral catheter placement is necessary.

Colpocleisis is a minimally invasive vaginal procedure that has regained attention in recent years as an appropriate option for elderly women who do not wish to preserve vaginal sexual function [7-11]. While several reports have shown that colpocleisis can improve lower urinary tract symptoms (LUTS), such as voiding difficulty and urinary urgency or frequency, its efficacy in treating urinary retention has not been adequately studied. In this retrospective study, we sought to investigate the clinical outcomes of colpocleisis in elderly women with POP-associated urinary retention, focusing on the postoperative improvement in the voiding function and procedural safety.

## Materials and methods

This study was approved by the Ethics Committee of Saga University Hospital (approval number: 2020-07-R-09). Informed consent was obtained through an opt-out process, wherein study details were made publicly available via institutional postings, and eligible participants were provided the opportunity to refuse participation.

We retrospectively reviewed the records of 165 patients who underwent surgical treatment for POP at our department between January 2019 and December 2024. Among them, 90 (55%) underwent laparoscopic sacrocolpopexy (LSC) or robot-assisted sacrocolpopexy (RASC), and 75 (45%) underwent colpocleisis. Colpocleisis was performed only after confirming that the patient had no history of vaginal intercourse and did not intend to engage in sexual activity in the future.

**Figure 1:**
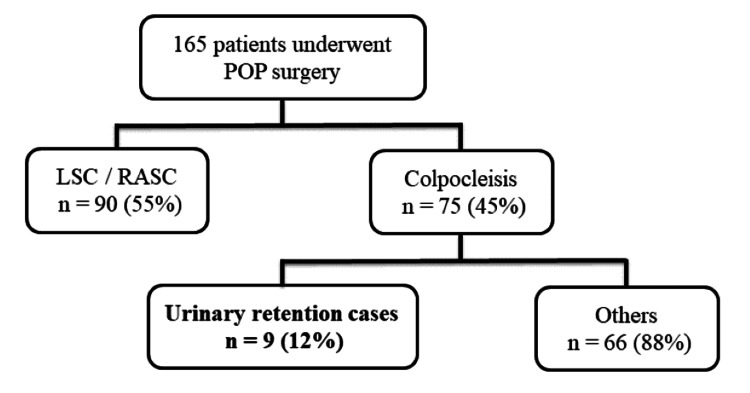
Distribution of POP surgical cases and preoperative urinary retention This flowchart illustrates the distribution of the 165 patients who underwent surgical treatment for POP at our institution. Of these, 90 (55%) underwent LSC or RASC, while 75 (45%) underwent colpocleisis. In the colporrhaphy group, nine patients (12%) presented with preoperative urinary retention POP: pelvic organ prolapse; LSC: laparoscopic sacrocolpopexy; RASC: robot-assisted sacrocolpopexy

Of the 75 patients who underwent colpocleisis, nine (12%) had preoperative urinary retention and were included in the present analysis (Figure 1). Urinary retention was defined as an inability to void spontaneously, necessitating either intermittent catheterization or continuous urethral catheter placement. These nine patients were selected based on the presence of persistent urinary retention from the time of referral. In all cases, patients were already managed with either an indwelling urethral catheter or intermittent self-catheterization before consultation at our institution due to prolonged voiding difficulty.

The following parameters were evaluated. First, patient characteristics were recorded, including age, BMI, parity, and comorbidities such as diabetes mellitus, prior pelvic surgery, spinal disorders, and pelvic organ prolapse quantification (POP-Q), assessed using the standardized system of the International Continence Society [12]. Second, surgical outcomes were analyzed, including operative time, estimated blood loss, intraoperative and postoperative complications, time to urethral catheter removal, and the length of postoperative hospital stay. Finally, the postoperative voiding function was assessed based on the voided volume per micturition, maximum urinary flow rate, and postvoid residual volume.

At our institution, patients scheduled to undergo POP surgery are routinely referred to the gynecology department for a preoperative evaluation, including transvaginal ultrasonography, cervical cytology (Pap smear), and an endometrial biopsy if indicated. If malignancy was ruled out, uterine preservation was chosen. If atypical cells or malignancies are detected in the cervix, transvaginal hysterectomy (TVH) is performed in collaboration with gynecologists.

Le Fort colpocleisis was performed for uterine-preserving cases or in patients with cervical abnormalities. The Le Fort procedure involves the partial preservation of the anterior and posterior vaginal wall mucosa, which is sutured together to close the vaginal canal. All patients underwent a preoperative urodynamic examination. During the pressure flow study, gauze was placed in the vaginal canal to reduce prolapse, and detrusor pressure (Pdet) was assessed in the corrected anatomical position. For urethral pressure profilometry, the gauze was removed, and urethral closure pressure was measured in the presence of prolapse.

## Results

The median age of the nine patients included in this study was 79 (range: 70-92) years, and the median BMI was 22.5 (range: 18.1-29.2) kg/m^2^. The median parity was two (range: one to three), and the median POP-Q stage was stage 3 (range: 2-4). Comorbidities included diabetes mellitus in four cases and spinal disorders in three cases (multiple myeloma with sacral tumor, spinal cord injury, and lumbar spinal canal stenosis). Two of the patients had a history of TVH (Table 1).

**Table 1:** Baseline characteristics of patients who underwent colpocleisis for POP with urinary retention *[12] This table summarizes the clinical characteristics of nine elderly women with POP and urinary retention who underwent colpocleisis. The median patient age was 79 years. Comorbidities included DM, multiple myeloma, spinal cord injury, lumbar spinal stenosis, and a history of TVH. POP-Q staging, determined according to the standardized system proposed by the International Continence Society [12], showed a median stage of 3. POP: pelvic organ prolapse; BMI, body mass index; DM: diabetes mellitus; TVH hx: history of transvaginal hysterectomy

No.	Age, years	BMI, kg/m²	Parity	Past history	POP-Q stage^*^
1	74	25.4	2	DM	3
2	78	18.1	1	Multiple myeloma	3
3	76	26.1	3	Spinal cord injury	2
4	79	20.8	3	Lumbar spinal stenosis	4
5	70	22.5	2	DM	3
6	91	22.1	1	None	3
7	87	29.2	3	TVH hx	3
8	81	24.9	2	DM	2
9	92	20.4	3	DM, TVH hx	3
Median	79	22.5	2		3

**Figure 2:**
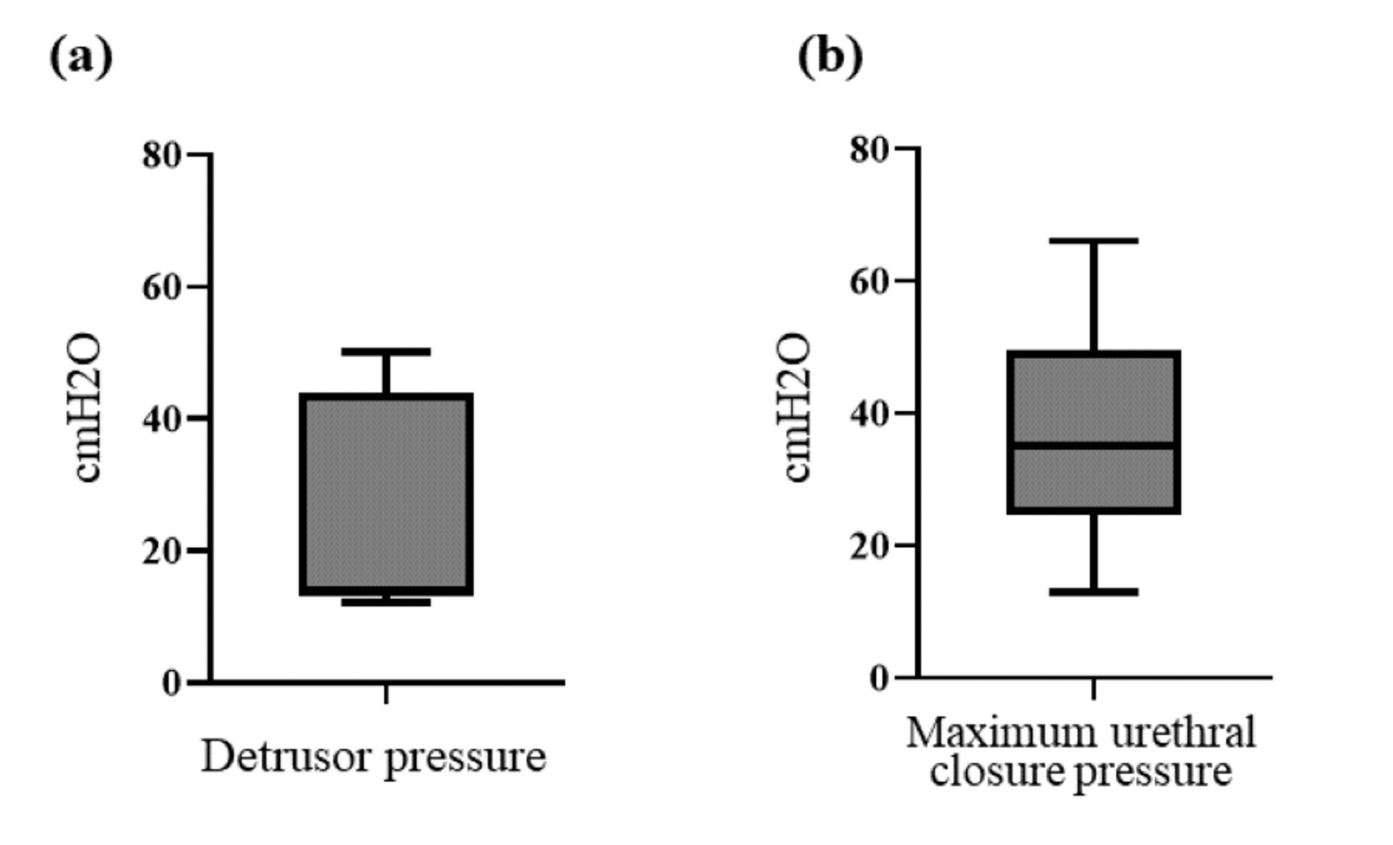
Preoperative urodynamic findings in patients with urinary retention (n = 9) Box plots show the distributions of (a) detrusor pressure and (b) maximum urethral closure pressure (MUCP). The median detrusor pressure was 14 cmH_2_O, and the median MUCP was 35 cmH_2_O

In preoperative urodynamic studies, four patients (Cases 1-4) were able to store urine but were unable to void, making detrusor pressure (Pdet) measurements unobtainable. In the remaining five patients who were able to void, the median Pdet was 14 (range: 12-50) cmH2O. The maximum urethral closure pressure (MUCP) was measurable in all cases, with a median of 35 (range: 13-66) cmH_2_O (Figure 2).

**Figure 3:**
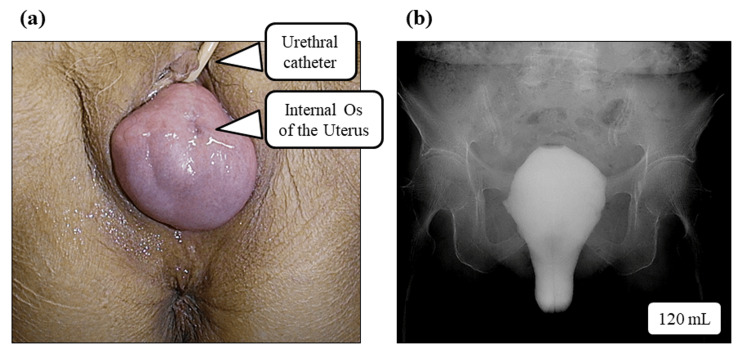
Representative case (No. 6) of pelvic organ prolapse with urinary retention (a) External genital view showing complete uterine prolapse with visible internal cervical os. (b) Cystography performed in the standing position after instillation of 120 mL contrast medium into the bladder in the supine position, demonstrating a large cystocele

A representative case of POP associated with urinary retention (Case 6) is shown in Figure 3. Figure 3a shows the preoperative external genital view demonstrating complete uterine prolapse with a visible internal cervical os. Figure 3b shows a cystogram obtained in the standing position after instilling 120 mL of contrast medium into the bladder in the supine position. A large cystocele is observed.

The surgical outcomes are summarized in Table 2. The median operative time was 115 (range: 34-180) minutes, and the median estimated blood loss was 0 (range: 0-175) mL. No intraoperative or postoperative complications were noted. The median time to urethral catheter removal was three (range: one to five) days, and the median postoperative hospital stay was six (range: 4-11) days. In two cases, hospital discharge was delayed due to COVID-19 infections among family members. The median follow-up duration after surgery was three (range: 1-18) months, and no POP recurrence was observed in any patient. In the three patients who underwent concurrent TVH, a pathological evaluation of the resected specimens revealed no malignancies, and no further gynecological follow-up or treatment was deemed necessary.

**Table 2:** Perioperative outcomes in elderly women undergoing colpocleisis for POP with urinary retention ^*^Concomitant transvaginal hysterectomy. ^†^Prolonged hospital stay due to COVID-19 in family members. This table summarizes the perioperative characteristics of the nine elderly patients who underwent colpocleisis for POP associated with urinary retention. The parameters included the patient age, operative time, estimated blood loss, perioperative complications, time to urethral catheter removal, and postoperative hospital stay. No perioperative complications were observed in any of the patients. Median values are shown in the bottom row POP: pelvic organ prolapse; COVID-19: coronavirus disease 2019

No.	Surgery time, minutes	Blood loss, ml	Complications	Time to urinary catheter removal, days	Postoperative hospital stay, days	Follow-up period, months
1	90	0	None	1	5	18
2	115	0	None	1	7	3
3	125	25	None	3	4	1
4^*^	129	0	None	3	6	1
5^*^	129	14	None	2	6	1
6	75	8	None	5	11^†^	5
7	78	10	None	3	11^†^	4
8^*^	180	175	None	3	6	5
9	34	0	None	2	8	1
Median	115	0	None	3	6	3

**Figure 4:**
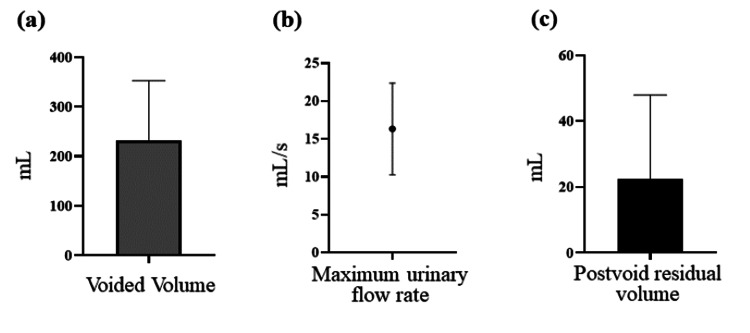
Postoperative voiding function in elderly women undergoing colpocleisis for POP with urinary retention (n = 9) The voiding function was assessed using uroflowmetry after catheter removal. Median values and interquartile ranges are presented for the following parameters. (a) Voided volume: bar graph. The median volume was 175 mL. (b) Maximum urinary flow rate: dot plot with interquartile error bars. The median value was 15.9 mL/s. (c) Postvoid residual volume: Bar graph. The median value was 20 mL. Error bars indicate the interquartile range for all panels POP: pelvic organ prolapse

Regarding the postoperative voiding function, eight patients (89%) regained spontaneous voiding and were successfully weaned from catheterization or intermittent catheterization. The remaining patient (Case 3), who had a neurogenic bladder due to spinal cord injury, was unable to void spontaneously, but self-catheterization became easier, leading to an improved quality of life. The postoperative voiding parameters were as follows: median voided volume per micturition: 175 (range: 120-420) mL; median maximum urinary flow rate: 15.9 mL/s (range: 10.5-22.6 mL/s); and median postvoid residual volume: 20 mL (range: 0-70 mL) (Figure 4).

## Discussion

This retrospective study investigated elderly women with advanced POP complicated by urinary retention who underwent colpocleisis. Eight of the nine patients regained spontaneous voiding postoperatively, and no perioperative complications or POP recurrence were observed in any case. Even in the remaining patient, who did not regain the ability to void spontaneously due to a neurogenic bladder associated with spinal cord injury, catheterization became easier, resulting in improved quality of life. These findings suggest that colpocleisis may be a safe and effective surgical option for elderly patients with POP presenting with urinary retention.

Urinary retention in women is relatively rare and most commonly caused by either DU or BOO. POP can lead to BOO by inducing anatomical changes, such as kinking or compression of the bladder neck or urethra, thereby causing voiding dysfunction [3,4]. Indeed, Abdel-Raheem et al. reported that voiding dysfunction occurs in up to 33% of women with advanced POP (stage ≥3) [5], which corresponds to the severity of the cases included in this study. In elderly women, voiding dysfunction may arise from multiple overlapping factors, including age-related decline in detrusor contractility as well as comorbidities such as diabetes and spinal disorders [5]. In the present study, preoperative urodynamic testing revealed a low detrusor pressure in many patients, suggesting that impaired detrusor contractility may have contributed to urinary retention.

While conservative treatments, such as pessary insertion, have been reported to improve voiding dysfunction in POP patients, their effectiveness may be limited in severe cases. Romanzi et al. found that patients with grade 3 or 4 cystoceles were more likely than those with grade 1 or 2 cystoceles to experience voiding difficulties and urethral overactivity, which could often be improved by pessary use [13]. However, severe POP cases are prone to pessary dislodgement, which limits their long-term success. In addition, self-catheterization may be considered for urinary retention, but this can be difficult in elderly patients due to reduced manual dexterity or cognitive impairment, resulting in long-term catheter dependence. In such patients, colpocleisis, which can be performed transvaginally and requires only a short operative time, may serve as a useful intervention for improving both voiding management and the quality of life.

Previous studies have reported that colpocleisis improves LUTS in terms of frequency and urgency [4,14,15]. However, its efficacy for urinary retention remains unclear. Our results suggest that anatomical correction of POP may relieve mechanical obstruction of the urethra and bladder neck, allowing the remaining detrusor function to support adequate voiding, thus highlighting the clinical relevance of this procedure. Colpocleisis offers several advantages: (1) it is a low-invasive transvaginal surgery with short operative time; (2) it involves native tissue repair, avoiding mesh-related complications; and (3) it is generally well-tolerated even by frail elderly patients [16-18]. This may be particularly advantageous for diabetic patients, who have a higher risk of infection and may benefit from non-mesh surgical options [19]. Therefore, for elderly women with no desire for future sexual activity, colpocleisis should be actively considered as a treatment option to improve the quality of life and prevent recurrence.

This study had several limitations. First, the analysis is based on a small number of cases from a single institution. Second, the relatively short postoperative observation period precludes conclusions regarding long-term outcomes, including voiding function and POP recurrence. Third, the retrospective design may introduce patient selection bias. Future prospective studies with larger cohorts and detailed urodynamic analyses are needed to validate these findings and explore how factors such as preoperative detrusor strength and the degree of BOO influence postoperative outcomes.

## Conclusions

In this small retrospective study of elderly women with POP-related urinary retention, colpocleisis was associated with short-term improvements in voiding function and no perioperative complications. While these findings suggest potential clinical utility, further prospective studies with larger cohorts are needed to confirm the long-term safety and efficacy of this procedure.

## References

[1] Nygaard I, Barber MD, Burgio KL (2008). Prevalence of symptomatic pelvic floor disorders in US women. JAMA.

[2] Wu JM, Vaughan CP, Goode PS, Redden DT, Burgio KL, Richter HE, Markland AD (2014). Prevalence and trends of symptomatic pelvic floor disorders in U.S. women. Obstet Gynecol.

[3] Slieker-ten Hove MC, Pool-Goudzwaard AL, Eijkemans MJ, Steegers-Theunissen RP, Burger CW, Vierhout ME (2009). The prevalence of pelvic organ prolapse symptoms and signs and their relation with bladder and bowel disorders in a general female population. Int Urogynecol J Pelvic Floor Dysfunct.

[4] Munno GM, La Verde M, Lettieri D (2023). Pelvic organ prolapse syndrome and lower urinary tract symptom update: what's new?. Healthcare (Basel).

[5] Abdel Raheem A, Madersbacher H (2013). Voiding dysfunction in women: how to manage it correctly. Arab J Urol.

[6] Mavrotas J, Gandhi A, Kalogianni V, Patel V, Batura D (2022). Acute urinary retention. Br J Hosp Med (Lond).

[7] Fitzgerald MP, Richter HE, Bradley CS (2008). Pelvic support, pelvic symptoms, and patient satisfaction after colpocleisis. Int Urogynecol J Pelvic Floor Dysfunct.

[8] Kurata S, Ichibagase Y, Nanri M, Noguchi M, Uozumi J (2015). The situation regarding colpocleisis as a surgical procedure for the repair of pelvic organ prolapse in our hospital. Nishinihon J Urol.

[9] Maeda A, Tobu S, Udo K, Noguchi M (2022). Female pelvic organ prolapse and female sexual function. Nishinihon J Urol.

[10] Pizzoferrato AC, Thuillier C, Vénara A (2023). Management of female pelvic organ prolapse-Summary of the 2021 HAS guidelines. J Gynecol Obstet Hum Reprod.

[11] Shalabna E, Cohen N, Assaf W, Zilberlicht A, Abramov Y (2025). Frailty and pelvic organ prolapse: Colpocleisis with or without hysterectomy as a treatment modality in elderly patients. Eur J Obstet Gynecol Reprod Biol.

[12] Bump RC, Mattiasson A, Bø K (1996). The standardization of terminology of female pelvic organ prolapse and pelvic floor dysfunction. Am J Obstet Gynecol.

[13] Romanzi LJ, Chaikin DC, Blaivas JG (1999). The effect of genital prolapse on voiding. J Urol.

[14] Lu YX, Hu ML, Wang WY (2010). Colpocleisis in elderly patients with severe pelvic organ prolapse (Article in Chinese). Zhonghua Fu Chan Ke Za Zhi.

[15] Wang Q, Lin H, Wu N, Li Y, Zhao R, Xu Y, Lin C (2024). Outcomes of a novel modified total colpocleisis for advanced pelvic organ prolapse in elderly women and its efficacy on lower urinary tract symptoms. Int J Gynaecol Obstet.

[16] Grzybowska ME, Futyma K, Kusiak A, Wydra DG (2022). Colpocleisis as an obliterative surgery for pelvic organ prolapse: is it still a viable option in the twenty-first century? Narrative review. Int Urogynecol J.

[17] Liang L, Ao S, Wang S (2024). Efficacy and safety of Le Fort colpocleisis in the treatment of stage III-IV pelvic organ prolapse. BMC Womens Health.

[18] Padoa A, Tomashev R, Yekutiel M, Hassouna H, Mendel L, Fligelman T (2025). Outcome of obliterative versus reconstructive surgery for pelvic organ prolapse in women of advanced age - a propensity score analysis. Eur J Obstet Gynecol Reprod Biol.

[19] Lu M, Zeng W, Ju R, Li S, Yang X (2021). Long-term clinical outcomes, recurrence, satisfaction, and regret after total colpocleisis with concomitant vaginal hysterectomy: a retrospective single-center study. Female Pelvic Med Reconstr Surg.

